# Possibilities of Influencing Procedural Pain Associated with Premature Newborn Retinopathy Screening with Oral Clonidine

**DOI:** 10.3390/children9111659

**Published:** 2022-10-29

**Authors:** Jiri Dusek, Eliska Simkova, Eva Fendrstatova, Radim J. Sram, Hana Kotouckova, Jan Voracek

**Affiliations:** 1 Neonatology Department, Ceske Budejovice Hospital, Bozeny Nemcove 54, 370 01 Ceske Budejovice, Czech Republic; 2 Faculty of Health and Social Sciences, University of South Bohemia, J. Boreckeho 1167, 370 11 Ceske Budejovice, Czech Republic; 3 Pediatrics Department, Ceske Budejovice Hospital, Bozeny Nemcove 54, 370 01 Ceske Budejovice, Czech Republic; 4 Institute of Experimental Medicine, Academy Sciences of the Czech Republic, Videnska 1083, 142 20 Prague, Czech Republic; 5 Department of Mathematics, College of Polytechnics, Tolsteho 16, 586 01 Jihlava, Czech Republic; 6 Faculty of Management, Prague University of Economics and Business, Jarosovska 1117/II, 377 01 Jindrichuv Hradec, Czech Republic

**Keywords:** clonidine, oxybuprocaine hydrochloride 0.4%, procedural pain, retinopathy in premature newborns

## Abstract

Background: The aim of our study was to compare the analgesic/sedative effects of various fundus-related procedural pain management strategies on the risk of retinopathy in premature infants. Method: This was a prospective comparative study involving a total of 94 neonates randomized to three groups meeting the criteria for at-risk neonates. Ophthalmologic screening was performed to evaluate the outcome of three procedural pain management strategies. The intensity of pain over time during and after the screening examination was evaluated. At the same time, we also looked at the occurrence of vegetative symptoms and their influence by the chosen medication. Pain response was observed in all 94 neonates enrolled in the study. In group A, no pain treatment was given. Group B had a local anesthetic oxybuprocaine hydrochloride 0.4% introduced into both eyes immediately prior to the examination. Group C received oral clonidine. The study was conducted as a pilot project and aimed to clarify the problem so that a project with a higher proband representation could take place in the future. Consequently, we performed quantitative analysis of complete pain and vegetative functions, followed by a qualitative analysis of their internal components. Results: In our study, we identified the most considerable effects for all three groups, including NIPS (Neonatal Infant Pain Scale) responses immediately during and after the examination. The influence of vegetative functions is of a longer-term nature and increased values can be clearly demonstrated even six hours after the examination. Conclusion: The current results identify and quantify differences among all three methods of pain treatment on the level of single variables. Their internal structures, however, can be analysed only qualitatively because of the small size of the analysed sample.

## 1. Introduction

The main research question in our study was to evaluate the analgesic effect of the three procedures in screening for retinopathy in preterm infants and to determine whether clonidine has a positive effect compared to the other methods we used. Another research question was to evaluate whether the administered medication and the performed examination influenced the vegetative function of the newborns and whether the chosen methods influenced the intensity of vegetative expression. A reasonably good analgesic effect was shown for cotton wool balls soaked in breast milk or 24% sucrose solution put in the mouth (1); favourable results were also achieved with the mere sucking of a pacifier or non-nutritious sucking (2). Our literary research identified Cochrane’s systematic review (3): the use of Clonidine as an analgesic/sedative in potentially painful examinations and procedures in the newborn. The authors found no references meeting their criteria recommending or warning against the use of clonidine in the prevention or treatment of procedural or postoperative pain or pain associated with other conditions in newborns (3). We decided to conduct a study evaluating the effect of clonidine in a specific indication (retinopathy testing in a premature newborn), which was not evaluated in the Cochrane review.

### 1.1. Procedural Pain: Definition

Procedural pain is defined as pain caused by painful stimuli during usual medical care. The period critical for the development of the fetal nervous system is the third trimester of pregnancy. In the preterm neonate, this is a period when the future infant is exposed to painful stimuli in the intensive and emergency care unit [1,2]. In preterm neonates, multiple painful stimuli may induce structural and physiological changes with permanent effects on their nervous system [3].

In a retinopathy screening, this is a specific type of procedural pain, where it is a combination of symptoms resulting from locally administered cycloplegia by means of parasympatholytics [4,5], muscarinic receptor blockers, locally administered drugs with sympathomimetic effect, and an alpha-1 adrenergic receptor agonist, as in our examination. At the same time, pressure is exerted on the bulb. This leads to an effect on the vegetative nervous system, with all the resulting consequences. We have also tried to focus our research on this aspect. 

### 1.2. Pain Scale

The system selected for the purpose of the present study was the well-known Neonatal Infant Pain Scale (NIPS) developed by Lawrence in 1993 [6]. The issue of using different pain assessment scales is addressed in the neonatal systematic review of randomized trials published in 2021 [7]. Here, the NIPS scale was ranked second in the use of pain assessment scales with 23% use in studies. The first-ranked scale was the Premature Infant Pain Profile or Premature Infant Pain Profile—Revised (48%) [7]. We opted for the NIPS scale because of its long-term use at our department and because of its good interpretability by all health care professionals [7].

### 1.3. Option in Pain Management

Pain management in the neonate is based on several methods. Currently, the main methods employed for the relief of procedural pain are non-pharmacological methods of pain management involving both behavioral and environmental measures. There was high-quality evidence for the beneficial effect of sucrose (24%) with non-nutritive sucking (pacifier dipped in sucrose) or 0.5 mL of sucrose orally in preterm and term infants. To evaluate the method using sucrose for analgesia in newborn infants undergoing painful procedures, we chose the Cochrane review (3), where the effect of this method is positively evaluated. However, to evaluate, it should be noted that it primarily concerns procedural pain in general, without taking into account the specificity of the eye investigation [8]. When using pharmacological means, we should be concerned with neonatal metabolism and, also, whether a term or preterm neonate is involved. Preterm neonates require lower baseline doses of a medicine and, also, longer intervals between the doses [9].

Oxybuprocaine hydrochloride 0.4% (Benoxi^®^) is an ocular local anesthetic, administered immediately prior to the screening examination. Oxybuprocaine hydrochloride reversibly blocks the propagation and conduction of nerve impulses along nerve axons, thus inducing temporary anesthesia without affecting pupil dilatation or accommodation of the eye. According to earlier studies, local anesthesia can be an effective tool in immediate pain relief: in this particular case, during insertion of the eye speculum; however, the effect is a short-term one, lasting only several dozens of seconds and hence not completely appropriate for the screening examination [10]. However, neither method appears to be effective when comparing the effectiveness of local anesthesia and the effect of glucose in a retinopathy screening examination. Nevertheless, the use of local anesthesia is mentioned in recommendations, e.g., in Sweden, but also in the Czech Republic and China [5,11,12].

Another option in procedural pain management in the neonate is the use of α-2 adrenergic agonists such as clonidine and dexmedetomidine. The broad spectrum of their actions includes central muscle relaxation, analgesia and anxiety relief [13,14]. Alpha-2 adrenergic receptors are spread throughout the central and peripheral nervous system, specifically in the pontine locus coeruleus, medullospinal tracts, rostral ventrolateral medulla, and the dorsal horn of the spinal cord. Alpha-2 agonist agents cause neuromodulation in these centers, leading to sedation, analgesia, vasodilatation, and bradycardia with little effect on the respiratory drive, which accounts for their good safety profile. The 2 major drugs in this group are Clonidine and dexmedetomidine. Their clinical applications in anesthesia practice include sedation in the intensive care unit or for minor procedures, adjuvant to general and regional anesthesia, analgesia, and as premedicating agents [15].

### 1.4. Retinopathy

Premature infants who met the following inclusion criteria based on the Guidelines for the Screening of Retinopathy of Prematurity in the Czech Republic issued by the Czech Medical Association Ophthalmology Branch [16,17] were considered eligible. Screening for retinopathy of prematurity (ROP) is performed in all neonates born before gestational week 32 and/or birth weight up to 1500 g, also including neonates with oxygen requirements higher than 0.6 FiO_2_ lasting more than 3 h. The screening examination is performed by the ophthalmologist using indirect ophthalmoscopy or a wide-angle digital RetCam system, with the conditio sine qua non being complete mydriasis. 

## 2. Method

### Description of Study Conduct

This was a prospective, comparative study randomizing 3 groups of neonates meeting the criteria for screening for ROP. Our study was designed to assess the pain neonates experience during the examination and whether or not the pain can be managed in any way. 

Those eligible for enrolment included neonates born at the hospital’s Neonatology Department as well as those born in another hospital within the district and subsequently transferred to our department because of their high-risk status.

The selection of probands into the test groups was performed in such a way that neither the ophthalmologist nor the assessment staff knew the setup of each group. Patients were always selected up to the required cumulative quantity in the test groups. One patient could also occur in more than one study group. The study was conducted between October 2020 and March 2021. Where applicable, the CONSORT 2010 statement [18] for the proper design and analysis of experiments was adhered.

Ethical Approval: The study was carried out according to the guidelines of the Helsinki Declaration and approved by the Ethics Committee of Ceske Budejovice Hospital, a.s. (Protocol Code 106/20 dated 23 October 2020). To enroll any neonate, all mothers signed their written consent.

Statistical analyses were performed using IBM SPSS Statistics 22 software (Armonk, NY, USA). Inductive-approach experiments were conducted using SAS Enterprise Miner v. 13.2 software (Cary, NC, USA) modules StatExplore, HP Cluster, Segment Profile, Variable Clustering and Link Analysis.

Group A included 29 neonates undergoing standard screening for ROP with usual care. The neonates in this group were instilled only with conventional mydriatic eye drops (phenylephrine 2.5% and homatropine 2.0%), one into either eye and another one 20 min later within an hour before the examination. 

Group B of neonates included 34 neonates. This group was instilled, in addition to usual care, with a drop of the local anesthetic (oxybuprocaine hydrochloride 0.4%; Benoxi^®^, Producer: UNIMED PHARMA s. r. o.) into either eye immediately prior to the examination, that is, just before eye speculum insertion. 

Group C, with 31 neonates, was administered oral clonidine (oral Magistraliter made by the pharmacy of the hospital Ceske Budejovice) at a dose of 2 μg/kg 30 min before the examination.

To standardize the screening examination, it was performed in all neonate groups identically with the ophthalmologist blinded to their allocation. The study was designed to evaluate changes in the NIPS pain scale [6] Table 1 and as additional separately assessed changes in vegetative parameters. Table 2. Data of all groups of neonates were recorded in the same manner at six pre-defined time points; assessment was started immediately before the screening examination to the end at 6 h later. The vegetative scores were performed on infants as described in Table 3 along the NIPS score. The maximum vegetative score is 6 points. It should be noted that the feeding times as well as visits by the attending ophthalmologist are standardized in our department, implying that all neonates were assessed at about the same times post-feeding. All data were collected at predefined time intervals, i.e., immediately before screening examination and during, immediately after, and at one, three and six hours afterwards.

## 3. Results

### 3.1. Statistical Analysis

#### 3.1.1. Characteristics of the Groups

In total, 94 neonates were enrolled into the study that met the criteria for ROP screening in the Czech Republic. For this number we decided that the groups would be sufficiently statistically evaluable and at the same time the study was carried out within 6 months. The youngest neonate was born at 24 ± 3 gestational weeks, so its gestationally corrected age was 31 ± 0 weeks at the time of screening. The oldest neonate was born at 40 ± 1 gestational weeks and underwent the examination at 42 ± 0 weeks of gestationally corrected age. More details are shown in Table 3.

#### 3.1.2. Data Characteristics

Homogeneity of groups A, B, and C in terms of medians of gestational age at birth, gestational age at screening examination and sex was tested using the parametric version of analysis of variance (ANOVA) and the Kruskal–Wallis test (nonparametric version of ANOVA). Neither of the tests showed a significant difference in gestational age at birth (groups A vs. B, A vs. C, *p* = 0.132 and *p* = 0.267, respectively) among the groups. Likewise, no significant differences were found in gestational age at examination (*p* = 0.806 and *p* = 0.739, respectively). Differences were demonstrated in sex (*p* = 0.031 and *p* = 0.033, respectively).

Homogeneity of the groups in terms of the equality of variances was assessed using Leven’s test. In both gestational age at birth and gestational age at examination, no significant difference was found between the variances in the individual groups (*p* = 0.871 and *p* = 0.270, respectively) whereas a difference was demonstrated for sex (*p* = 0.000). Overall, the groups were homogeneous in their gestational ages at birth and screening examination, and non-homogeneous in sex, resulting in exclusion of this variable. During the frequency analysis the variables characterizing birth and exam ages were also removed because of their similarly sized and ordered additive influence on all groups.

All data were collected at predefined time intervals, i.e., immediately before screening examination and during, immediately after, and at one, three and six hours afterwards. The corresponding variables were coded with the related prefixes (NIPS_, VEG_), followed by temporal characteristics (BEFORE, DURING, AFTER_0, AFTER_1 and AFTER_6). For different kinds of analyses, their ranges were considered either as continuous or discrete.

### 3.2. Groups Structure

**Figure 1:**
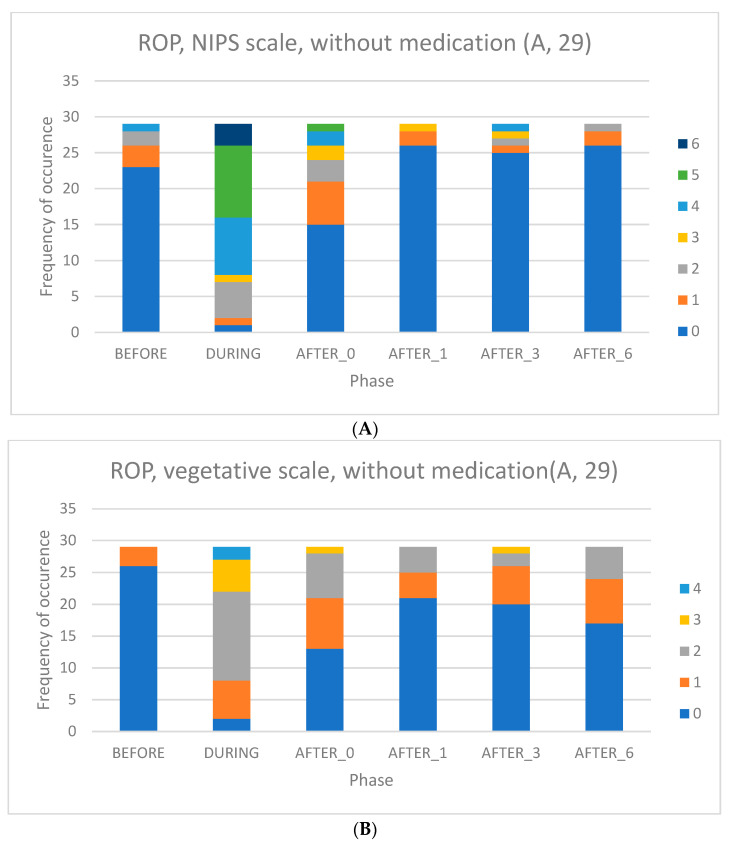
(**A**) Group A (except for standard mydriatic eye drops as usual care)—NIPS scale. (**B**) Group A (except for standard mydriatic eye drops as usual care)—vegetative symptoms.

**Figure 2:**
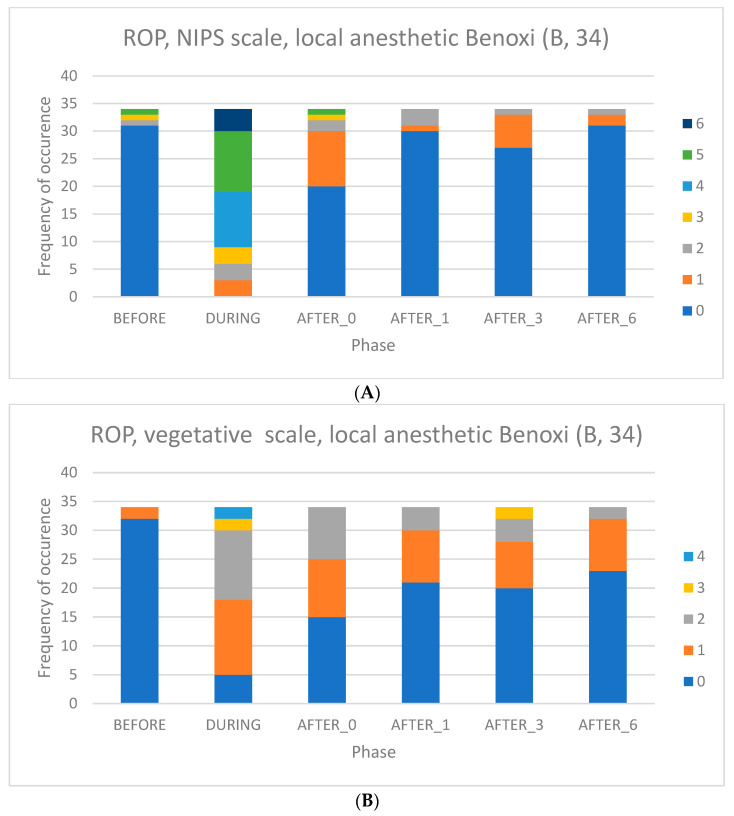
(**A**) Group B instilled the local anesthetic oxybuprocaine hydrochloride 0.4% NIPS scale. (**B**) Group B instilled the local anesthetic oxybuprocaine hydrochloride 0.4%—vegetative symptoms.

**Figure 3:**
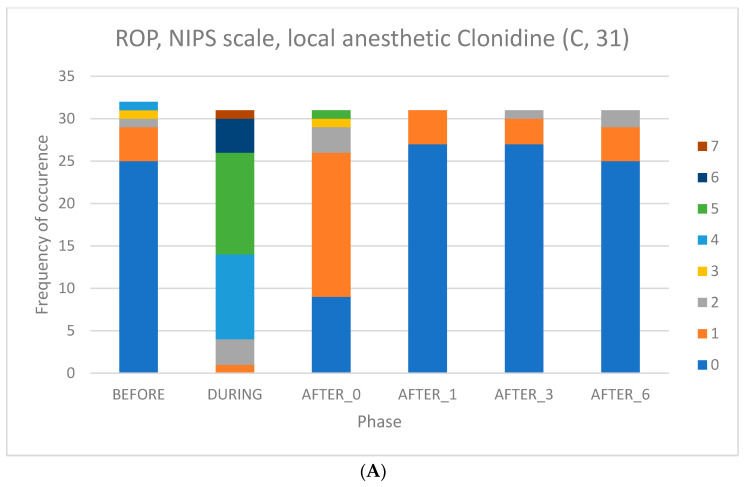

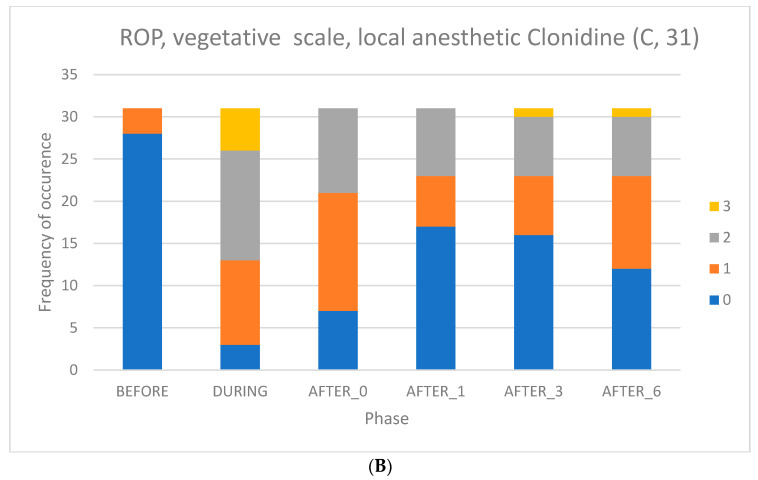
(**A**) Group C receiving the local anesthetic clonidine (oral)—NIPS scale. (**B**) Group C receiving the local anesthetic clonidine (oral)—NIPS scale.

The contingency graphs of the analysed groups can be found in Figure 1A,B, Figure 2A,B and Figure 3A,B, where all data according to the above criteria are captured, before, during and after the examination.

In group A, without pain treatment, Figure 1A is NIPS scale and Figure 1B shows a shell of vegetative symptoms in this group.

In group B, Figure 2A demonstrates that use of a local anesthetic (oxybuprocaine hydrochloride 0.4%; Benoxi^®^) shows affect to the intensity of pain experienced by the neonate during the examination or immediately thereafter, or in the longer term. Figure 2B shows a shell of vegetative symptoms scale in group B.

In group C in Figure 3A, the assessment of oral administration of clonidine at a dose of 2 μg/kg is 30 min before screening—NIPS scale. Figure 3B shows a shell of vegetative symptoms in this group.

### 3.3. Comparison, Statistical Analysis, Levels of Significance

A comparison of the figures shows there are no large differences among the groups; in fact, the differences are almost negligible. All neonates undergoing screening for ROP in our department during the study period did experience pain during and immediately after the examination without a marked beneficial effect of any of the tested interventions, i.e., usual care with mydriatic eye drops, local anesthesia with oxybuprocaine hydrochloride 0.4% or oral clonidine.

Statistical analysis of pain perception among groups A (only usual care), B (oxybuprocaine hydrochloride 0.4%, a local anesthetic) and C (Clonidine, a local anesthetic) was performed using parametric analysis of variance (ANOVA) and its non-parametric alternative, the Kruskal–Wallis test. Neither of these two tests demonstrated a significant difference both in pain intensity and occurrence of vegetative symptoms in any of six temporal examinations, i.e., all obtained *p*-values were greater than 0.05.

Inductive algorithms, however, were able to find some worthwhile differences among the study groups, indistinguishable for deductive approaches. Initially, the segmentation of the complete dataset with respect to single groups was performed. This analysis found the most relevant variables, distinguishing segments A, B and C from the population. In the next step, internal categories, i.e., corresponding NIPS and VEG scales of hereby identified variables were examined with frequency analysis and quantified using chi-square (χ) metrics. For the final discussion, only categories including more than five elements and having χ values at least two times higher than variable average were selected. The achieved results are summarized in Table 4. Inductive experiments were conducted by the StatExplore and Segment Profile nodes of SAS Enterprise Miner.

## 4. Discussion

Our study is the first with published results to evaluate the efficacy of clonidine in this procedural examination in premature newborns. It is also the first to evaluate vegetative symptoms along with the NIPS pain scale. There is an upcoming Swedish multicentre study in the available literature to evaluate the efficacy of clonidine in ROP testing. The data are not yet available and it will be interesting to compare it with the data we have collected [19].

In the study, clonidine was selected as a drug for potential treatment of pain at screening in group C. In the relevant literature, clonidine has multiple, more- or less-used effects. This was mainly due to its action in the cholinergic and serotonergic pathways, resulting in an analgesic effect (28), so we decided to apply it in the third screening group (group C).

Our study is the first with published results to evaluate the efficacy of this drug in this procedural examination in premature newborns. 

However, in this particular group (group C), we did not achieve unambiguous positive results, but neither did we in the other groups studied. The whole set is limited by the size of the examinations performed. However, it can be said that in our study group neither of these groups has clear positive results that would lead to the conclusion that the medication reduces pain or vegetative manifestations. Other potential areas of use of clonidine based on its anti-inflammatory effect or shortening the duration of opioid treatment have not been investigated in this study. Our research is currently correlated with study recommendations that do not recommend routine administration of clonidine due to background screening. Similarly, the positive effect of a local anaesthetic is not confirmed. At the same time, we demonstrated the safety of use of clonidine as there were no serious complications when administered in the endpoints. An important point for reflection is the effect of administered local medication on cycloplegia, which is a possible reason for higher occurrence of vegetative symptoms as well as stimulation of the eyeball.

We demonstrated the safety of the use of clonidine as there were no serious complications when administered in the endpoints.

The suppression of painful symptoms, including vegetative manifestations such as bradycardia, impaired food tolerance and apneic pauses, is important for clinical practice, and their effect should be further investigated. The study was conducted as a pilot project and aimed to clarify the problem so that a project with a higher proband representation could take place in the future.

## 5. Conclusions

From our research, we cannot recommend clonidine in the indication of procedural pain relief in ROP examination as the desired effect has not been achieved.

An important finding is the higher frequency of vegetative symptoms that persist for a longer time compared to the increased NIPS pain scale, which is higher during screening but adjusts rapidly to baseline values.

## Figures and Tables

**Table 1:** NIPS score.

	Pain Assessment	Score
**Facial Expression**		
0—Relaxed Muscles	Restful face, neutral expression	
1—Grimace	Tight facial muscles, furrowed brow, chin, jaw ( negative facial expression—nose, mouth brow)	
**Cry**		
0—No cry	Quiet, not crying	
1—Whimper	Mild moaning, intermittent	
2—Vigororous cry	Loud scream, rising, shrill, continous ( Note: silent cry, may be scored if baby is intubated by evidence by obvious mouth and facial movment)	
**Breathing Pattern**		
0—Relaxed	Usual pattern for this infant	
1—Change in breathing	Indrawing, irregular, faster than usual, gagging, breath holding	
**Arms**		
0—relaxed/Restrained	No muscular rigidity, occasional random movements of arm	
1—flexed/extended	Tense, straight arms, rigid and/or rapid extension, flexion	
**Legs**		
0—relaxed/Restrained	No muscular rigidity, occasional random movements of legs	
1—flexed/extended	Tense, straight legs, rigid and/or rapid extension, flexion	
**State of Arousal**		
0—Sleaping/Awake	Quiet, peaceful, sleeping or alert, random legs movements	
1—Fussy	Alertrestless and thrashing	

**Table 2:** Vegetative score.

	Difference betwen Curent Saturation (dSatO2) < 10%	dSatO2 10–20%	dSatO2 > 20%
points	0	1	2
	heart rate (beats’min) difference (Hrd) < 10%	Hrd 10–20%	Hrd > 20%
points	0	1	2
	Apneic episode (AE) NO	AE Yes	
points	0	1	
	Food residuum in the stomach (FR) < 30%	FR > 30%	
points	0	1	

**Table 3:** Characteristics of neonates.

	Group A	Group B	Group C
Mean gestational age at birth, weeks	27.2	28.2	28.7
Mean gestationally corrected age at screening, weeks	35.3	34.9	35.1
Sex, males/females, number	23/6	28/6	31/0

**Table 4:** Sets of distinguishing variables for study groups ordered according to their logworth > 0.01. Empty cells in category characteristics section did not fulfil the selection criteria. Effect L/H means that the real occurrence in category was lower or higher than expected in case of independency.

Group	Variable Characteristics		Category Characteristics		
	Name	Group Logworth	Scale Grade	χ Contribution	Effect
A	NIPS_AFTER_0	0.025	1	12%	L
	NIPS_AFTER_3	0.018			
	VEG_DURING	0.017			
B	VEG_AFTER_6	0.024	2	22%	L
	NIPS_BEFORE	0.021			
	NIPS_AFTER_0	0.017			
	NIPS_AFTER_3	0.016	1	21%	H
	VEG_DURING	0.015			
C	NIPS_AFTER_0	0.041	0	15%	L
			1	24%	H
	VEG_AFTER_6	0.028	0	18%	L
	VEG_AFTER_0	0.021	0	26%	L
	VEG_AFTER_1	0.012	2	34%	H
	NIPS_DURING	0.012			
	NIPS_BEFORE	0.010			

## Data Availability

The datasets used and/or analyzed in the present study are available from the corresponding author upon reasonable request.
